# Editorial: New tumor immune checkpoints and their applications in tumor immunotherapy

**DOI:** 10.3389/fmolb.2026.1873649

**Published:** 2026-05-20

**Authors:** Kai Zhang, Yang Mi, Bing Yang

**Affiliations:** 1 Department of Medical Laboratory, Zhengzhou University Third Affiliated Hospital, Zhengzhou, China; 2 Henan Key Laboratory of Helicobacter Pylori, Microbiota and Gastrointestinal Cancers, Marshall Medical Research Center, Fifth Affiliated Hospital of Zhengzhou University, Zhengzhou, China; 3 Department of Medical and Nursing Science, International College, Krirk University, Bangkok, Thailand; 4 Department of Cell Biology, College of Basic Medical Sciences, Tianjin Medical University, Tianjin, China

**Keywords:** immune checkpoint inhibitors, immune subtypes, NK cell, resistance mechanisms, t cells, tumor immune checkpoints, tumor immunotherapy

## Introduction

More than a century ago, William Coley observed that bacterial products could trigger tumor regression, a finding that laid the foundation for cancer immunotherapy despite the era’s limited immunological knowledge. Today, we continue to pursue the same fundamental goal: equipping the immune system to recognize and eradicate malignant cells. The advent of immune checkpoint inhibitors (ICIs) targeting PD-1/PD-L1 and CTLA-4 has profoundly reshaped the therapeutic landscape for numerous malignancies. Yet durable clinical benefit remains limited to a subset of patients, and both primary and acquired resistance pose enduring challenges. This has spurred an intensive search for novel immune checkpoints and robust predictive biomarkers to enable more precise and effective immunotherapeutic strategies. Beyond the thoroughly characterized PD-1 and CTLA-4 axes, a new generation of immune modulators—such as TIGIT, LAG-3, TIM-3, VISTA, and B7-H3—has emerged as critical regulators of T-cell activation and exhaustion, while metabolic and proteolytic pathways are increasingly recognized for their role in shaping the tumor immune microenvironment.

Against this backdrop, this Research Topic in *Frontiers in Molecular Biosciences* assembles a Research Topic of 13 articles—spanning original research, reviews, case reports, and methodological innovations—that collectively advance our understanding of emerging immunoregulatory pathways and their translational potential. The contributions encompass diverse tumor types and apply a wide spectrum of approaches, from multi-omics bioinformatics and Mendelian randomization to prospective clinical observation and deep learning. We structure this editorial around three thematic pillars: novel immune checkpoint molecules and mechanistic insights, predictive biomarkers and resistance mechanisms, and clinical evaluation of ICIs and treatment-associated toxicity.

## Novel immune checkpoint molecules and mechanistic insights

The identification of new immune regulatory molecules is central to expanding the immunotherapeutic armamentarium. A comprehensive review by Liu et al. highlights human leukocyte antigen G (HLA-G), a non-classical MHC class I molecule that facilitates immune escape by inhibiting NK cell cytotoxicity, impairing antigen-presenting cell function, and inducing regulatory T cells. The review positions HLA-G as a promising next-generation checkpoint target. Extending the scope to metabolic immunomodulation, Tian et al. employ Mendelian randomization to identify adenosine deaminase (ADA) as a potential risk factor for esophageal cancer and systematically link ADA isoforms to distinct tumor-infiltrating immune cell profiles, also exploring Traditional Chinese Medicine interventions that might modulate ADA-related pathways. In the context of proteolytic regulation, Qu et al. perform longitudinal profiling of serum ADAM17 in multiple myeloma and reveal its dynamic association with T cell alterations, underscoring ADAM17 as a possible biomarker of immune dysfunction and disease progression. At the interface of T-cell biology and immunotherapy, Wei et al. demonstrate how antigen-specific T cell precursor frequency in tumor-draining lymph nodes governs the proliferation of stem-like T cells, offering mechanistic insights into durable anti-tumor immunity with direct implications for adoptive cell therapy and vaccine design. Together, these studies illustrate that a network of classical and non-classical immune checkpoints—along with the metabolic and proteolytic circuits that support them—constitutes a rich terrain for therapeutic intervention.

## Predictive biomarkers, immune subtypes, and resistance mechanisms

Precision immunotherapy demands refined stratification strategies and biological understanding of resistance. Wang et al. define cuproptosis-related gene signatures that classify gastric cancer into distinct immune subtypes with divergent prognoses, introducing this recently recognized form of regulated cell death as a framework for assessing therapeutic vulnerability. In non-small cell lung cancer, Shrestha et al. tackle the clinically challenging KEAP1/STK11-mutant subset, demonstrating that the mutant-associated redox phenotype suppresses STING and MDA5 expression, attenuates interferon signaling, and fosters a T cell-exclusion microenvironment. These findings not only clarify the molecular underpinnings of an immunotherapy-refractory genotype but also identify nodes for rational combination therapy. The growing incorporation of artificial intelligence into biomarker development is exemplified by Yu et al., who present a deep-learning-based classifier for head and neck cancer detection using digitized whole-slide histology images, highlighting the rapidly evolving intersection of computational pathology and immuno-oncology. Complementing high-dimensional molecular approaches, Cho et al. report a prospective observational study showing that temporal changes in tongue color during ICI therapy in non-small-cell lung cancer—quantified through digital tongue diagnosis derived from East Asian medicine—may offer a low-cost, non-invasive adjunct for real-time monitoring. Collectively, these works reflect an increasing emphasis on multi-parametric biomarkers and integrative algorithms that promise to better match patients to appropriate immunotherapies.

## Clinical evaluation and treatment-associated toxicity

Real-world evidence continues to illuminate both the efficacy and safety profile of ICIs. Liberko and Soumarova describe a case of complete remission achieved with nivolumab in a young patient bearing an advanced MSI-H rectal cancer, contextualizing this exceptional response within the broader literature on PD-1 blockade for mismatch repair-deficient tumors and reinforcing the predictive value of microsatellite instability. Xie et al. report significant tumor regression and quality-of-life improvement following combined treatment with the anti-PD-1 antibody tislelizumab and capecitabine in a patient with extensive perianal Bowen’s disease, broadening the therapeutic spectrum of ICIs to include cutaneous premalignancies not amenable to local therapies. A comprehensive review by Wang and Wang further synthesizes current mechanisms, clinical advances, and persistent challenges of immunotherapy in gastric cancer, providing a panoramic view of how ICIs are being integrated into gastrointestinal oncology. With respect to safety, Schellhammer et al. retrospectively analyze 164 cancer patients receiving ICIs and find no significant changes in liver attenuation or liver enzyme levels, offering reassuring evidence that these agents may not exacerbate hepatic steatosis or subclinical liver injury—a finding particularly relevant for managing patients with pre-existing metabolic liver disease. These clinical vignettes and systematic reviews underscore the need for a multidimensional evaluation framework that incorporates baseline patient characteristics, dynamic biomarker monitoring, and vigilant assessment of immune-related adverse events.

## Future perspectives

The articles collected in this Research Topic illustrate the breadth and depth of current investigation into novel tumor immune checkpoints and their applications. Looking ahead, several priorities warrant attention. The functional overlap and context-dependent expression of immune regulatory molecules call for systematic combinatorial strategies grounded in mechanistic understanding, potentially pairing novel checkpoint blockade with epigenetic modulators, metabolic inhibitors, or rational vaccine platforms. Translating emerging targets from preclinical validation to clinical deployment will require coordinated efforts, innovative trial designs, and the integration of integrative multi-omics approaches—from genomics and transcriptomics to single-cell analyses—to delineate the interplay among tumor metabolism, immune regulation, and therapeutic response. The continued incorporation of artificial intelligence into diagnostic and predictive pipelines is poised to accelerate progress across these domains. We extend our sincere appreciation to all the authors, reviewers, and the editorial team for their contributions, and hope this Research Topic will serve as a catalyst for further investigation, interdisciplinary collaboration, and ultimately the advancement of effective, personalized immunotherapy for patients with cancer.

